# Girl-only clubs’ influence on SRH knowledge, HIV risk reduction, and negative SRH outcomes among very young adolescent girls in rural Malawi

**DOI:** 10.1186/s12889-021-10874-x

**Published:** 2021-04-27

**Authors:** Wanangwa Chimwaza Manda, Nanlesta Pilgrim, Mphatso Kamndaya, Sanyukta Mathur, Yandisa Sikweyiya

**Affiliations:** 1 School of Public Health, University of the Witwatersrand, Johannesburg, South Africa; 2 Centre for Reproductive Health, College of Medicine, University of Malawi, Blantyre, Malawi; 3 Independent consultant, Washington, DC USA; 4 School of Applied Sciences, University of Malawi-The Polytechnic, Blantyre, Malawi; 5 Population Council, Washington, DC USA; 6 Gender and Health Research Unit, South African Medical Research Council, Pretoria, South Africa

**Keywords:** Sexual and reproductive health, Very young adolescents, HIV risk, Girl-only club

## Abstract

**Background:**

Early adolescence is an important period to lay the foundation for positive sexual health development that can overcome sexual and reproductive health (SRH) challenges faced by very young adolescents (VYAs) as they reach puberty and sexual debut. In this study, we explored the following questions: first, what are the experiences of VYA girls on DREAMS’ Go Girl club participation? Second, how does club participation influence the VYAs SRH knowledge to reduce their risk for HIV and negative sexual health outcomes?

**Methods:**

This was a qualitative study in which twenty-three in-depth interviews were conducted with VYA girls aged 12–14 years. These girls were enrolled in girl-only clubs in two rural southern districts in Malawi. The clubs were a part of larger comprehensive HIV prevention project called DREAMS (**D**etermined, **R**esilient, **E**mpowered, **A**IDS-free, **M**entored, and **S**afe) which provided an evidence-based core package of interventions to VYAs to prevent HIV. Interventions included improved access to key health services, education support, social skills, asset building, and economic strengthening. Narrative inquiry was used to generate first-hand accounts of the girls’ experiences with club participation. Thematic analysis was used to generate themes from the transcribed stories.

**Results:**

Six main themes were generated: 1) reasons for joining the clubs with desire to learn about SRH as a motivation for joining the clubs.; 2) influence on gender norms and roles whereby participants described a change of gender roles and norms at home; 3) influence on child abuse practices whereby participants reported a decline in child abusive practices at home;4) influence on life skills and social networks whereby participants described learning about networking; 5) support to go back to school whereby out-of-school girls described how economic empowerment of their guardians facilitated their return to school; and 6) influence of clubs on SRH knowledge acquisition and behaviours whereby participants described acquiring knowledge on sexual health issues.

**Conclusion:**

Girls-only HIV and SRH programs coupled with economic empowerment for their families can be effective in keeping VYA girls in school and improving SRH knowledge and health seeking behavior.

**Supplementary Information:**

The online version contains supplementary material available at 10.1186/s12889-021-10874-x.

## Background

UNICEF categorizes adolescents as very young adolescents (VYAs) ages 10 to 14 and older adolescents ages 15 to 19 [1]. There are about 1.2 billion adolescents globally, half of whom are VYAs [2]. Adolescents face a myriad of challenges related to their sexual and reproductive health (SRH). In low and middle income countries (LMICs), where the bulk of VYAs live and bear the heaviest burden of SRH challenges, investment in positive youth development to promote SRH is increasing [3]. However, most of the adolescent SRH interventions focus on older adolescents, overlooking the SRH needs and concerns of VYAs [2–5]. This oversight is because most of VYAs are considered less sexually active when compared with older adolescents [3, 6]. On the contrary, some studies have reported that a significant number of VYA girls and boys have had unsafe sex [7]. Furthermore, VYAs have unique sexual health needs that require support. For instance, VYAs are at a stage where their sexual curiosities have just begun [2, 5, 7]. They also experience enormous biological, cognitive, sexual, and emotional and social changes during this period [3, 6]. These changes include development of secondary sexual characteristics such as breast budding and menstruation in girls, ejaculation in boys, and a majority of girls and boys begin to experience sexual arousal [3, 6]. In addition, they begin to form new gender roles and sexual identities within their social contexts. As these developments are occurring, VYAs also acquire information, develop attitudes and experiment with behaviors and relationships [6, 8, 9]. These developments and experiences predispose them to SRH challenges such as early unintended pregnancies and STI infections including HIV [10].

A recent review conducted by Bruce and Chong revealed that in most youth SRH programs, clients tend to be older youth (often 20 + years old) rather than VYAs [11]. In addition, most adolescent SRH programs reported gaps such as lack of adequate attention to gender norms and protective social factors such as involving parents, teachers and neighborhoods; lack of safe spaces within community institutions; and failure to build skills for safe transition into later adolescence and adulthood [6]. Despite these challenges in the field of VYAs, globally there has been an increase in both research and programming in the past 5 years [2, 4, 12–15]. However, gaps still exist and more research is needed to inform VYA programs [6, 16].

Like most countries globally, a few adolescent health programs exist in Malawi that reach VYAs with SRH information, life and relationship skills, and violence prevention education; key elements considered to reduce HIV risk (e.g., delayed sexual debut) and empower girls [17]. For those reached by such programs, little is known about the experiences of VYAs in such programs and the impact of the programs on their lives [17]. As VYAs are a unique age group with specific needs that might be different from those of older adolescents, exploring their experience of participating in various SRH intervention programs is critical for designing programs that are culturally and age appropriate for VYAs.

The DREAMS initiative included VYA girls in health interventions implemented in two rural southern districts in Malawi. DREAMS is an initiative funded by the US government President’s Emergency Plan for AIDS Relief (PEPFAR) that aims to ensure that adolescent girls and young women (AGYW) have an opportunity to live **D**etermined, **R**esilient, **E**mpowered **A**IDS-free, **M**entored, and **S**afe lives. It aims to significantly reduce new HIV infections among AGYW by 40% in 10 countries, including Malawi [18]. Among the interventions is the Go Girl Club initiative which is being implemented by One Community project in Zomba and Machinga districts. Evidence from the implementation science that sought to measure the impact of the initiative among AGYW aged between 15 and 24 showed a positive impact where there were significant improvements in comprehensive knowledge about HIV, condoms, and prevention of mother-to-child transmission; significantly higher levels of support for equitable gender norms and higher relationship power among AGYW; fewer experiences of STI symptoms, and increased use of HIV testing and post-gender-based violence care; and there was a decrease in reported sexual violence from partners and non-partners among AGYW overtime [19]. In this paper, we report on first-hand accounts of experiences of VYA girls aged between 10 and 14 who participated in Go Girl clubs, and the clubs influence on their SRH knowledge to reduce the risk for HIV and negative sexual health outcomes.

## Methods

### Research design

This study was nested in a larger implementation science study (IS) that aimed to assess the implementation of the DREAMS initiative, including outreach and retention in the programs and HIV risk reduction among AGYW and male partners in Machinga and Zomba districts, Malawi [19]. In the DREAMS project, the AGYW who ranged between 10 and 24 years, were recruited to participate in girl-only clubs called Go Girl clubs. A central principle of DREAMs is the layering of interventions whereby AGYW were targeted with a range of interventions. These interventions were delivered through United States Agency for International Development (USAID) implementing partners. In the clubs, young women called club facilitators used a DREAMS tool kit which is a form of curriculum to train the participants in various skills relating to their sexual and reproductive health. In addition, the clubs served as a safe space for AGYW to learn how to prevent HIV including improved access to key health services, education support, social skills, asset building, and economic strengthening. While the main focus of the initiative is on the target group of 15 to 24 AGYW, the program is also targeting 10–14-year-old girls (see Table 1).

**Table 1:** Classification of Go Girl Club DREAMS Interventions

	The DREAMS GO! Girl Club Intervention Classification		
	10–14 years	15–19 years	20–24 years
**Primary Individual Interventions**	-Social asset building -HIV testing services -Condom information -Screen for case management	-Social asset Building -HIV testing services -Condoms -Access to contraceptive -Information and services	-Social asset building -HIV testing services -Condoms -Access to contraceptive -Information and services
**Secondary Individual Interventions**	-Combination of socio-economic approaches (VSL) for caregivers -Food security/ nutrition -Post violence care -Access to contraceptive information and services -Back to School Support	-Combination of socio-economic approaches (VSL) -Food security/ nutrition -Post violence care -Back to school support -Parenting skills -Case management	-Combination socio-economic approaches (VSL) -Food security/nutrition -Post violence care -Back to school support -Parenting skills

The DREAMS project was a two-year project which was implemented from 2016 to 2018. The IS study was conducted by the University of Malawi, College of Medicine (COM) in collaboration with the Population Council (PC) of the United States of America (USA) between July 2017 and May 2019. The target population of the IS study was adolescent girls and women between the ages of 15 and 24, whereas this study targeted VYA between the ages of 10 and 14.

This was a qualitative study which explored the experiences of VYA girls’ club participation from a postmodern social constructionist perspective [20–22]. This perspective asserts the subjective meanings of experiences, and that these meanings are varied and multiple, enabling the researcher to look for the complexity of views rather than narrowing meanings into a few categories or ideas [20]. As a result, the stories people tell are informed by the social and cultural environment in which they are embedded [22]. The study design and methods for this study were informed by this perspective. In addition, the Social ecological model (SEM) developed by Bronfenbrenner in 1979 underpinned this study [23–26]. The model looks at a person’s (e.g. child) development within the context of the system of relationships that form his or her environment [24]. Bronfenbrenner categorized the environment in which the person interacts into levels. The first level is the microsystem which is the environment nearest to the individual and includes psychological characteristics and behaviours [27]. The second level is the mesosystem this includes the family, relational and community spheres [27]. The third level is the Exosystem includes characteristics of the society at large (e.g. socio-economic status, healthcare policies, media, and gender) [27]. The forth category is the Macrosystem which the comprises of cultural values, customs, and laws [28–30]. The model has been used to understand adolescents’ health by identifying determinants of their health located at the individual level as well as those located in the community, structural and socio-physical environments [31–34]. The assumption behind using this model in this study is that the stories that VYA girls will tell about club participation will be affected by various factors at the different levels of the SEM.

In-depth interviews (IDIs) with VYA girls aged 12–14 were used to collect data. A narrative inquiry approach was followed in which unique stories of the study participants were synthesized [35]. This approach enables an exploration of how individuals interpret their daily experience at the same time looking at how they make use of the wider social/cultural resources to make sense of their lives [36]. An advantage of using this method with VYAs is that its story telling nature helps in rapport building, allowing participants to tell stories in their own way and to focus on key issues that are important to them [37]. In this study, narrative inquiry was used to explore VYA girls’ experiences of club participation and how it influenced their SRH knowledge to reduce their HIV risk and negative health outcomes.

### Study sites

This study was conducted in two rural southern districts of Zomba and Machinga in Malawi where the DREAMS project was being implemented. Machinga and Zomba districts have a total population of 735,438 and 851,737 respectively [38]. In both districts, one in three inhabitants are youth between the ages of 10 and 24 [38]. AGYW represent half of the youth in both districts making a total of 117,835 in Machinga and 141,158 in Zomba [38].

The districts were purposefully selected for the DREAMS project due to high HIV prevalence (13 and 16.3% among 15–49 year olds, in Zomba and Machinga respectively), HIV treatment gaps, high proportions of orphans and vulnerable children, high prevalence of early sexual initiation, high rates of childbearing during the teen years, and high school drop-out rates among girls [19].

In Zomba, the DREAMS project was implemented around the catchment area of two health facilities of Thondwe Health Centre and Namikango Mission Hospital. The catchment area consisted of a total of 63 villages. There were 88 Go Girl clubs in these villages in which a total of 1003 AGYW were enrolled. Only two out of 88 Go Girl Clubs included VYAs with a total of 20 VYAGs.

In Machinga, the Dreams project was implemented around the catchment area of Machinga District Hospital. The catchment area consisted of a total of 45 villages in which there were 75 Go Girl clubs. There was only one Go Girl club with a total of 10 VYAGs.

### Sampling and data collection

Participants were recruited using purposive sampling [20]. The study participants were beneficiaries of a DREAMS project and were between the ages of 10 and 14. At the time of interviews participants had been club participants for about 12 months. The inclusion criteria for participating in the study were availability and willingness to participate, ability to communicate experiences and opinions in an articulate, expressive, and reflective manner. Using the help of a project person working in the DREAMS projects, participants were identified and approached to ask for their participation in the study. Only VYA girls between the ages of 12 and 14 were finally selected, as we could not find any VYA girls below age 12 who met our inclusion criteria.

Twenty-three IDIs were conducted by the first author. Seven interviews were conducted with VYAs from the VYA club in Machinga, the remaining three club members did not agree to participate. In each of the two clubs in Zomba, eight interviews were conducted with VYAs and two club members from each of the two clubs were not present to participate. Taking into consideration that participants in this study were very young girls, the interviews were done by the first author who is a female researcher with experience of working with adolescents. Interviews were conducted in Chichewa, a dominant spoken language in the study setting. The interviews were conducted face to face in the community where the participants lived. To ensure privacy and confidentially a private house was arranged within the community where individual interviews took place. No one else was present during the interview apart from the interviewer and the participant. All interviews were recorded using a digital recorder. A semi-structured interview guide (Additional file 1) was used to collect narratives on experiences of club participation and how this had influenced their SRH knowledge and health. Two research questions were explored in this study: 1) what are the perceptions and experiences of VYA girls on club participation? and 2) how does the club participation influence the VYAs’ SRH knowledge to reduce their risk for HIV and negative sexual health outcomes? The narrative inquiry first started with questions that aimed to build rapport while also gathering some information about the participants. These included questions about time use and mobility, social support and networks. The second part of the inquiry focused on asking participants to share stories on club participation experiences including how and why they joined and how the clubs have been useful to them. All interviews were once-off. Each interview lasted between 30 and 40 min. All participants were still club members at the time of the interview. The narrative interview approach was chosen in order to create a setting that encouraged and stimulated the participants to tell a story about their experiences from their perspectives as directly as possible.

### Data analysis

The audio-recorded IDIs were transcribed verbatim. Transcripts were then carefully translated from Chichewa into English without losing the original meanings. The transcription and translation were done by a hired experienced research assistant. To check for quality, the first author read the transcripts and checked the transcripts against the audios. The transcripts were then imported into the NVIVO software. A thematic analysis approach was used to identify themes within the data. The first author read the transcripts repeatedly to familiarize her-self with the content. A codebook was developed where codes and code definitions were made based on the objectives of this study. Codes were grouped into categories, then developed into themes, and analyzed using the Nvivo 11 software. Another independent researcher was engaged in coding to test the codebook to assess if there was inter-coder agreement [20, 39]. Quotes illustrating meaning or key message from the analysis were selected based on how best they represented the key message.

### Ethical considerations

Ethical approval to conduct this study was obtained from the College of Medicine Research Ethics Committee (COMREC) in Malawi (Ethics Approval Number: PP.01/17/2095), and from the University of the Witwatersrand Human Research Committee (HREC) in South Africa (Approval number: M181009) and from Population Council in the United States of America (Approval number: 784). Guidelines related to interviewing children and adolescents were followed to ensure that the study was conducted ethically, and that the procedures followed by the study protected the participants and minimized harm [40]. Before engaging the VYA girls a parent or guardian was approached to obtain her/his permission to interview the girl using an appropriate informed consent document. After parent/guardian consent to the VYA girl participation, the VYA girl was approached to provide assent.

## Results

### Social demographic characteristics of study participants

A total of 23 VYA girls were interviewed (Table 2). The age of the participants ranged between 12 and 14 13. The majority of the participants (78%) were in primary school and most of them were living with both parents.

**Table 2:** Social Demographic Characteristics of Participants

Characteristic	Number (***N*** = 23)	%
**Age**		
12	5	22
13	9	39
14	9	39
**Education Status**		
In School (Primary)	18	78
Out of School	5	22
**Housing (Living with)**		
Both parents	15	65
Single parent	5	22
Other (relatives)	3	13

### Reasons for joining the go girl clubs

The main reason for joining the clubs reported by almost every participant was the desire to learn about various issues concerning their lives. These issues varied amongst participants and included SRH, child abuse, and education.

*“I wanted to find out where I can go to report if my parents abuse me. And I also wanted to know how I can prevent getting HIV/AIDs.”* **IDI 1, Zomba**

Other participants especially the out of school girls described joining the clubs as an opportunity to do something with their time since they were not engaged in any activities at home:

“*When I had stopped going to school, because I had nothing to do at home, when it was morning; the sun would set while I was at home... So, when this organization came, our treasurer is the one who came to pick us saying ‘there is an organization, don’t just stay here at home. What you would have been learning at school, you will be learning here at the club. So not to waste time, this organization wants children between 10 up to, he said 14*.” **IDI 2, Machinga**

Apart from personal reasons to join Go Girls Club, most participants reported that the decision to join the clubs was influenced by their community leaders, teachers, neighbors, friends, and parents.

*“I was told about the Go Girls Club by my teacher who is also a chief. He told us that we should start coming here for club activities...*” **IDI 5 , Zomba**

“*I started in September. I joined because of my friends, right? A lot of them, right? were saying ‘let’s go and join, let’s join.’ So, I said ‘I will not join, because I do not know what I will learn there. I stopped going to school a long time ago. So if I should be going there, what will I learn?’ And they said, ‘go, go’ so I went.*” **IDI 3, Machinga**

Although the participants reported various reasons for joining the clubs, there was a general impression in their responses that a majority liked attending the clubs. This was also noted when participants were asked to report on the challenges that they encountered regarding club participation. Interviews revealed few challenges reported by the club participants. For instance, several participants reported that some VYAs in the community who had not joined the Go Girl clubs discouraged them from joining and participating in the clubs. These non-club members were reported to have said negative things about the Go Girls clubs, and this mostly stemmed from misconceptions about the clubs. The following extracts are illustrative:

“*Now us, many people were discouraging us saying ‘don’t go there, it is satanic.’ But us, we did not care, we went there so that we could learn. So what made me to join the Go Girl club is learning*...” **IDI 11, Zomba**

“*That time we were going they used to say, ‘you are busy going to Go Girl Club but you don’t receive anything there.’ So, we did not pay them attention. We just went*.” **IDI 4′ Zomba**

“*Most of our friends do not like to join this group, we don’t know why really. Those who like the program are few.... Some of them say the program encourages sex, and even when you tell them that there is a lot that you would avoid, they do not take it. They believe that this encourages a lot of girls to have sex or have unprotected sex*.” **IDI 3, Machinga**

### Influence on gender norms and roles

Learning about gender norms was a notable experience that was reported by several participants. Several participants narrated experiences whereby most of the household chores at their respective homes were done by them (girls) whilst their brothers were left to play. A notable experience was the participants’ increased ability to explain what they had learned at the clubs to their parents and siblings and being able to effect change without negative repercussions. For instance, most participants reported that after learning at the clubs that there is no difference between what a male and female can do, they communicated this learning to their parents and siblings, and that now they share chores with male siblings.

“...*also that at home my brother was not doing any house chores and after learning about gender I explained it to my brother that he doesn’t have to differentiate work between men and women. And he started working for example, when I cook in the afternoon he cooks in the evening, and he also fetches water.*” **IDI 9, Zomba**

*“We learnt that there is no difference between boys and girls, girls can do what boys do… it changed me because I explained to my parents that a boy and a girl can do similar work. We now do similar work with my brothers.”* **IDI 7, Zomba**

### Influence on child abuse practices

Another notable experience that was reported as a result of club participation was a decline in child abuse practices at home. Participants said they learned about child abuse practices such as parents overworking children, girls missing school in order to take care of younger siblings, and girls being forced to get married. Upon learning at the clubs that these practices were abusive, some participants engaged their parents and explained to them why and how such practices are abusive. These participants reported that they noted a gradual decrease in these abusive practices at home. Participants attributed the decrease to the knowledge they acquired at the club and transferred to their parents and guardians. One participant narrated a story about her father who used to mistreat her whenever she misbehaved by beating her, not giving her food and making her sleep outside their home. She indicated that she reported this to her club facilitator who intervened by talking with her parents. In her own words:

*“As for me, the problems that I meet is that my father beats me and one day they made me sleep outside, so I told them [club facilitators] ... For example, one day they told me to sweep the compound and that day I said that I am tired, someone else should do it today, so they didn’t give me food and beat me. So, I told them [club facilitators] and they came to speak with my parents.”* **IDI 7, Zomba**

Another girl narrated a similar history of abuse at home and how this changed after attending the club:

*“We were also being abused by parents but ever since we joined Go Girl clubs they stopped because we explained to them everything we learned from one community (Project running the Go Girl clubs)* ...*They teach us examples of child abuse like overworking a child, forcing girls into marriage, leaving children to take care of babies, and absenting us from school... Like making a girl baby sit her siblings without going to school”* **IDI 5, Zomba**

### Influence on life skills and social networks

The majority of participants reported experiencing a change in their behaviors because of the important life skills they learned at the clubs. Several participants reported better use of their time compared to the period before they joined the clubs. Other participants said they learned about networking with other girls. For these participants, attending the clubs enabled them to know other girls by their names, where they came from and reportedly developed a bond and ‘sisterhood’ that made them to stand up for one another in times of trouble. Other lessons included learning about the importance of working as a team or in unity:

*“At first before joining one community when we were back from school, we were not doing household chores but rather just playing but now we do household chores and thereafter do our studies.”* **IDI 6, Zomba**

*“I have learnt also about how to strengthen the club... Being people who are united… (silent for some time), because people who can be united, do great things together.”* **IDI 3, Zomba**

*“they said that we should know each other; for instance, me and my friends we should know each other so my friend needs to introduce herself, then the rest of us will do the same thing by mentioning our names too.... They said that the advantage of knowing each other is that if your friend is involved in an accident then you can be able to assist her that is if you know her. Yes*.” **IDI 1, Machinga**

### Support to go back to school

In the interviews, several participants reported that they had at some point stopped attending school. Our data revealed no differences among girls who had dropped out of school in terms of whether they lived with parents, lived with single parents, or lived with relatives. For instance, there were participants who had never dropped out of school despite living with a single parent or a relative. Participants who had dropped out of school indicated they had done so because of poverty which caused them to lack basic needs such as clothes and school materials like books, school uniform, and writing materials. However, participants reported that the organization (One Community) which had introduced the clubs also introduced Village Savings Loans (VSLs) for their parents or other relatives. These VSLs are income generating activities whereby women contribute monetary shares into one basket and give each other loans to start up small scale business. Some participants reported that through these VSLs their parents or guardians were able to meet their school needs, resulting in them returning to school. Other participants returned to school because some clubs distributed school learning materials and uniforms.

*“Me, through the Go Girl Club, village banks were introduced in our village, my mother managed to join the village bank. She borrowed money from there and do business. The profits from the business are used to get me necessities for my school.”* **IDI 5, Zomba**

“[in the club] *we received school bags, pencils, erasers, notebooks, uniforms and we also got tested for HIV.*” **IDI 7, Machinga**

However, there were several participants who had decided to drop out of school because they did not see a future in their studies, and therefore chose to stay home. These participants reported that at the Go Girl Club they listened to motivational talks given by girls who had worked hard at school despite facing similar challenges as them, including poverty, and made it in life. They said through the motivational talks they learned about the importance of education in their lives and decided to back to school.

“*I have benefited from Go Girl Club that I did not really know the reason for my going to school, but I have known now. Through Go Girl Club I have learnt that it is up to you to choose which job you want after finishing school. So, I was convinced, I went to continue with school.”* **IDI 8, Machinga**

*“For example, I had stopped going to school. But when I started learning at Go Girls, I continued with school and they encouraged us about school. And also, I learnt about my dreams that ‘it is up to you to decide which job to work in the future when you finish school.’ So I would like that when am done with my school, I should be a supervisor in the One Community Project.”* **IDI 8, Zomba**

### Influence on sexual and reproductive health knowledge and behaviors

The majority of the participants described knowledge acquisition as a critical part of their experience in the clubs. Most of the participants reported learning about sexual health issues such as HIV, contraception, menstrual hygiene, and STIs for the first time at the club. On these issues, participants narrated they had learned what HIV and AIDS are, how one would get infected with HIV, and how one should protect themselves from HIV, STIs and pregnancy by using condoms. While other participants indicated that they had prior knowledge about sexual health issues from school, they also indicated that at the club they had acquired in-depth knowledge about these issues.

“*I have benefited, right? Because I did not know that us; pregnancy how can we avoid it? Or AIDS how can we avoid it? We did not know. And also that people do cleanliness when they are on their monthly periods, we did not know; most of us; right? Because we learnt that while we were young. So we learnt it there at One Community.*” **IDI 2 Machinga**

*“The difference is that at this other place [school] they hide some of the issues from us while at the Go Girls club they tell us everything...Maybe the teachers hide these issues from us because we are still young, that is why they chose to hide the issues from us.”* **IDI 3, Zomba**

Apart from acquiring knowledge, some of participants reported application of this knowledge in their lives. A total of five participants said they were in sexual relationships. Except for one participant who was 13 years old, the rest of those who were sexually active were 14 years old. All five participants who were in sexual relationships reported condom use during sex.

“*We learnt a lot on sex issues for example, I wouldn’t have known that when having sex with my boyfriend I should use a condom. I also learnt that if you have plain [unprotected] sex you might get pregnant.*” **IDI 7, Machinga**

In addition, some of the participants reported testing for HIV. These participants included both those who were in sexual relationships and those who were not. Participants reported that through the clubs they were linked to HIV testing services. Interviews suggest that this linkage to HIV testing happened at the community level where club participants were referred to mobile/outreach clinics to access HIV testing services. Furthermore, participants also reported a linkage to HIV testing at the health facility where there was a project person through which the clubs could book an appointment for the club participants to access a service.

*“I got tested. They assisted us with accessing HIV testing services. Yes, they (mobile/outreach clinics) come here to provide us with the HIV testing services*” **IDI 4, Machinga**

*“At that time, they told us that if one wants to get tested we should go to the hospital there is One community personnel so if you make an appointment with…. you will just have to meet that person.”* **IDI 6, Zomba**

## Discussion

The aim of this study was to gather first-hand accounts of experiences of VYA girls who participated in Go Girl clubs, and explore the clubs’ influence on their SRH knowledge to reduce the risk for HIV and negative sexual health outcomes. Generally, all participants reported to have had a positive experience with participating in the clubs. Our findings suggest that VYA girls’ club participation experiences were affected by an interplay of factors at various levels of the SEM. For instance, at microsystem level, our findings suggest that the desire to learn more about sexual health issues was the main reason most participants joined the clubs. The findings also show that at mesosystem level, parents had influenced some VYA girls to join the clubs. This finding highlights the important role that parental support could play in influencing VYA girls to participate in programs that could have a positive impact on their health. On the other hand, our findings highlight the club participation’s influence on VYA girls knowledge, which is an individual level factor. Knowledge acquisition about health issues was reported to be a key benefit derived from participating in the clubs. This finding is consistent with literature showing that developmentally, young adolescents are at a stage where curiosity about their sexuality and sexual health issues is heightened [7]. However, it is also at this stage in life where misinformation about these issues abounds from peers, siblings, and other unreliable sources of information [3, 12, 14, 41]. It is therefore an important finding that the clubs provided a forum where VYA girls could access accurate sexual health information. This is a significant finding because in one study, lack of comprehensive knowledge about HIV in Zomba and Machinga districts was one of the key defining characteristics of HIV vulnerability among out-of-school AGYW [42]. This finding is also relevant because in the rural setting where this study took place; there are few places that provide accurate and comprehensive sex education to VYAs. Furthermore, VYAs in our study included out-of-school girls with no access to in-school sex education. Therefore, increasing avenues such as Go Girl clubs where VYA can access correct information about sexual health issues is critical for developing healthy sexual well-being of VYAs in this setting.

Furthermore, at microsystem level, the VYA girls were empowered with knowledge about gender norms and roles which enabled them to successfully challenge discriminatory gender norms at home. For instance, some participant’s narrated stories about equally sharing household chores at home with their brothers as something that resulted from their questioning of these norms. The finding that some VYA girls influenced a change in gender norms at their homes highlights the significance of family support, which is a critical factor at mesosystem level, in providing an enabling environment for VYA girls to implement what they learned from the clubs. Literature documents that gender norms and stereotypes are internalized and solidified during early adolescence and that these affect and drive SRH and well-being of individuals during the adolescence stage and later on in life [3, 43]. Findings from another study in the same setting conducted among AGYW indicated that low support for gender equitable norms was associated with higher HIV vulnerability among AGYW [42]. In the same study, it was found that AGYW with a high-vulnerability profile had significantly higher odds of HIV related outcomes (e.g., very early sexual debut, transactional sex, sexual violence from partners). Therefore, investing in programs that instill positive gender norms in VYAs has the potential to reduce HIV vulnerability among AGYW. Such programs can yield SRH and gender dividends that deal with negative gender norms way before they develop and take root in individuals.

Our findings suggest that, at mesosystem level, the Go Girl Clubs facilitated peer support and social networks among VYAS. In low and middle income countries, most adolescent girls in rural settings lack strong social networks due to gender bias which result in girls having less opportunities than boys to socialize and engage in leisure activities and income generating activities [3]. In such settings, interventions that build social skills have been documented to reduce social isolation in girls through providing them social safety nets through mentors and peers [3]. In this study, we found that the Go Girl clubs provided a forum for girls to connect, learn, and be inspired by one another. We also found that the Go Girl clubs provided an environment where the girls could meet and interact freely to discuss sexual health issues which was reported as a benefit by the participants. This finding is important as adolescent girls and young women in Machinga and Zomba districts face issues of social isolation and gender based violence [44, 45]. Thus, creating a forum for girls to talk about sexual health issues provide them with a rare opportunity to break the silence which usually surrounds issues of gender-based violence.

Our findings further show that participation in the Go Girl Clubs had another important impact at the mesosystem level as some parents of the VYA girls were economically empowered to be able to enroll their out of school VYA girls at school. The participants reported that their caregivers, specifically women, were supported by clubs in income generating activities which empowered them to provide for the girls. This finding is important considering that removing economic barriers and keeping girls in school is one of key strategies of reducing vulnerabilities to HIV infection among girls [46]. Empirical evidence suggests that keeping girls in school does not only reduce their vulnerabilities to HIV infection but has the potential to create a critical mass of healthy, educated, and financially independent women who make well-informed choices about their lives, including family planning [47, 48]. In rural areas such as where this study was conducted, early marriages, and HIV prevalence is high [38]. More programs such as the Go Girl Club interventions that encourage girls to stay in school are beneficial and should be expanded.

At exosystem level, our findings suggest that VYAs were able to access HIV testing services provided by the DREAMS implementing partners and government. Our findings show that the Go Girl clubs provided an avenue for young adolescent girls to be linked to health services. We have also shown that, through their participation in the Go Girl Clubs, several girls were linked to HIV testing services and tested. Furthermore, we have demonstrated that the girls learned where to report child abuse and sexual violence, if they experienced it. Considering their young age, most young adolescents are very unlikely to seek or access services in formal avenues such as health centers or via available community structures [3, 4]. Yet our findings highlight the important role the Go Girl clubs played in enhancing VYA girls’ self-efficacy and agency to seek or access HIV testing services.

Girl only clubs have also been implemented in countries such as Uganda, Nepal, Ethiopia, Egypt and Ghana with findings similar to those reported in this paper [49–51]. While girl-centered approaches like girl only clubs are not entirely new approaches, this study brings in findings from an implementation science study with a unique focus on VYA girls. Of great importance is the unique DREAMS approach which seeks to reduce HIV risk and increase agency of AGYW using a layered approach [18]. The layering of interventions is a fundamental principle of DREAMS which demonstrates that addressing multiple needs of young people will have greater impact on risk behaviors than any single intervention [18]. At the individual level, layering is defined as providing multiple interventions from the DREAMS core package to each DREAMS recipient [18]. In line with the SEM we observed that the DREAMS layered approach sought to address factors exposing VYA girls to HIV risk at individual level but also beyond this level as we have discussed above. Our findings complement findings from other DREAMS implementation science studies in other countries which have shown a positive impact of the layering approach in reducing risk for HIV among AGYW [52–54]. For instance in a study in Uganda, the DREAMS activities were found to reinforce and expand knowledge and contributed to positive behaviour change such as pregnancy delay, avoidance of HIV and reduction in risky behaviours that lid to HIV and pregnancy among AGYW ages 15–19 [52]. A quantitative study is Lesotho which compared AGYW who had participated in the DREAMS interventions with those who did not found out that the DREAMS interventions appeared to be effective at reducing overall levels of sexual risk among participants of all ages [53]. In this study we have shown how this approach through the girl-only clubs had an influence on SRH knowledge among VYA girls who participated in the clubs, and also how it influenced some of the social, economic, and cultural norms and practices that predispose AGYW to the risk for HIV. These findings are especially critical for VYA girls when the ‘SRH and gender’ dividend is taken into consideration whereby intervening at this age to strengthen their social and protective assets, will enable them to protect themselves from negative sexual health outcomes including reducing their risk for HIV.

### Limitations

This study only explored the experiences of the VYA girls attending the clubs. We did not assess participants’ knowledge, relationship with parents, and their HIV risk prior to them joining the clubs. As such, our findings are limited in assessing the effect of the clubs on these issues. Future studies would do well to collect baseline data on the girls’ experiences before attending the clubs. Another limitation is that we did not explore other stakeholders’ perspectives, such as club facilitators, parents and other family relations to the club participants. Furthermore, since the participants were still club members at the time of interview, there is a possibility of social desirability in that they might have been overly positive about the Go Girl clubs. In addition, the study conducted once off interviews which cannot assess longer term impacts on club participants. In this qualitative study, we interviewed VYAs who were purposively selected because of their participation in the Go Girl Clubs, our findings are not generalizable, however we hope that the narrative accounts and insights we gathered are of interests to other similar settings. In addition though our research questions were asking participants to narrate stories of their experiences about club participation it is possible that other DREAMS activities that were happening in the study area at the same time as the clubs may have influenced the participants’ views on the clubs.

## Conclusion

This study has highlighted the significance of delivering targeted multiple interventions at various levels of a SEM through girl-only clubs. Through club participation, the club members not only acquired comprehensive HIV/AIDS knowledge but were also able to access HIV testing services. Furthermore, participants who had dropped out of school returned to school. Thus, the study has shown that creating an enabling environment for VYAs to interact, learn, and discuss issues is one of the solutions to equip them with knowledge and skills to protect themselves from HIV and other negative sexual health outcomes later in life. In addition, the study has shown that girls-only HIV prevention programs coupled with economic empowerment for their families can be effective in helping out-of-school VYAs return to school and improve their HIV and SRH knowledge.

## Supplementary Information

**Additional file 1:.** In-depth Interview Guide

## Data Availability

The interview data of this study are available from the corresponding author upon reasonable request.

## Abbreviations

IDI: In-depth interview
SRH: Sexual and reproductive health
VYA: Very young adolescents
