# Supplementary material for: Girl-only clubs’ influence on SRH knowledge, HIV risk reduction, and negative SRH outcomes among very young adolescent girls in rural Malawi
Source: BMC Public Health. 2021 Apr 27;21:806. doi: 10.1186/s12889-021-10874-x (PMC8077750; doi:10.1186/s12889-021-10874-x)
Supplement: Supplementary file 1 — Additional file 1:. In-depth Interview Guide [file 12889_2021_10874_MOESM1_ESM.docx]

## Appendix 1: In-depth Interview Guide for Very Young Adolescent Girls (10-14)

**Social Support and Sexual Health among Very Young adolescent girls in two Districts in Malawi: A Narrative Inquiry**

**Profile of the IDI participants**

**District:**

**Age:**

**Lives with Parent or Not:**

**Marital Status:**

**Number of children:**

**Highest level of education completed:**

**Introductory script:**

*"My name is Wanangwa Manda. I am a student at the University of Witwatersrand, School of Public Health and the University of Malawi, College of Medicine, Blantyre Malaw. I like to thank you for taking time to participate in this interview. The aim of this study is to describe your experiences and opinions about your life as an adolescent girl. More specifically about how you seek help and support about your health and needs from others in this community. The interview will take about 30 and 60 minutes.*

*A few ground rules about this interview. I want you to do most of the talking. There is no right or wrong answer to the questions. I want to hear your opinions on different things that I will ask you. Your experiences and opinions are important. All your responses will be kept confidential. You do not need to answer any questions that you do not feel comfortable with.* *The study is voluntary, and you have the right to withdraw from participation at any point with no negative consequences.*

*Do you have any questions before I begin?"*

1. **Time use and Mobility**
2. In a regular day of the week where do girls like you spend most of time?
3. What do young girls like you do in your free time?
4. What responsibilities do young girls like you have at home?
5. Which spaces/places do most of the girls like you spend their time?

Probes

- - How safe are these spaces?
  - Tell me of a day or time when you were in any of this spaces and you felt insecure

1. **Social Network and Support**
2. Who do you often spend most of the time with or often chat with?
3. What activities do you do with them?
4. What issues do you discuss with them?
5. Tell me of a time when you faced a challenge or had a problem and when you wanted to tell someone?
6. Probes

- Who did you tell?
- Why did you choose this particular person?

1. **Parent or Guardian Support**
2. Where do you live?
3. Do you like it where you live?

Probes

- - If yes, what do you like about living there?
  - If not, what do you not like about living there?

1. Do you find it safe where you live? Why/why not?

Probes

- Tell me of a time when you felt it was not safe here?

1. Tell me who do you live with?

Probes

- - Have you lived with these people all your life?
    1. If not, how long have you been living with these people?
  - What role does each of these people play in your life?

1. Tell me about how comfortable you feel talking about issues in your daily life with people that you live with?

Probes

- - Tell about a situation or an issue in your life that you talked with the people you live with?
  - How did it make you feel talking to them?
  - If you have never spoken about issues, why didn’t you do so?

1. **HIV/AIDs and Other Reproductive Health Problems**
2. Tell me what you know about HIV/AIDs?
3. Tell me about the first time you heard about HIV/AIDs?

Probes

- Where was it?
- Who talked about it?
- What questions did you have?
- Did you ask questions
- Where else did you hear about HIV/AIDS

1. Tell me about a time when you discussed about HIV/AIDS with anyone?

Probes

- - Who did you discuss with?
  - What did you discuss about HIV/AIDS?
  - Do you think girls your age is at risk for HIV/AIDS? Why or why not?
  - Do you have any other specific questions or issues that you would want to know about HIV/AIDs?
  - Who would you love to talk with and why?

1. Tell me about the first time you heard about pregnancy

Probes

- Where was it?
- Who talked about it?
- What questions did you have?
- Did you ask questions
- Where else did you hear about pregnancy?
- Have you ever discussed with anyone about how girls can get or prevent pregnancy?
- Who did you discuss with?
- What did you discuss about?
- Do you have any other specific questions or issues that you would want to know about pregnancy?
- Who would you love to talk with and why?

1. Have you noticed any of the following signs of growing up in your body? (growing of hair in armpits or pubic region, development of breasts, beginning of menses)

Probes

- Tell about what happened when you noticed any of the above?
- What did you do?
- Who did you talk with about it?
- If you haven’t noticed any of these growing up signs yet, have you ever talked or heard about it somewhere?
- Tell about when and where you first heard about it?
- Do you have any other specific questions or issues that you would want to know about the signs of growing up that show in your body?
- Who would you love to talk with and why?

1. **For Married girls or girls with children**
   1. Have you ever had discussions about child birth or pregnancy related issues with anyone for example about antenatal, delivery and post-natal issues?

Probes

- Who did you discuss with?
- What did you discuss?
  1. Do you have any other specific questions or issues that you would want to know about these issues?

Probes

- Who would you love to talk with and why?

1. **Now let’s talk about girl and boy relationships**
2. Tell me about the first time when you heard about boy/girl relationship?
3. How common in this village that a girl your age would have boyfriend?
4. What are the reasons or motivations for having such relationships?
5. Tell me about a time when you had discussed about the boy/girl relationship issue with parents or guardians?

Probes

- What did you discuss?

1. Tell me about a time when you had discussed about the boy/girl relationship with friend or peers?

- What did you discuss?

1. Do you have any specific things about girl/boy relationships that you desire to learn about?

Probes

- What are the issues?
- Who would you want to discuss these things with?

1. **Enrolment and recruitment (this section aims to gather in-depth insights into participants’** knowledge of DREAMS, recruitment and enrolment procedures, and reasons for staying/dropping out of the program)

a. How did you come to know about the DREAMS program?

b. Can you please describe how you were selected to participate in the DREAMS program? *(Probes: Who approached you and where? What did they tell you about DREAMS? What services were you told were going to be provided?)*

c. Why do you think you were eligible to participate in the DREAMS program?

d. What motivated you to join the program?

1. *Experience with DREAMS service/training sessions****(Club Participation)***
2. How long have you been part of the DREAMS program?
   *(Probe: When were you approached to join the DREAMS program?)*
3. We have heard that the DREAMS program has many different services. Can you tell me more about them?
4. Which services have you been participating in?
   For each of the services that the respondent has been participating in, ask the following questions:

- Where was this service provided?
- How often did you receive this service?
- Who usually provides this service?
- Describe their attitude, behaviour of the service provider? How they made you feel?
- Please explain if you felt this service were useful to you.
- Please explain things about this service that you did not like or were not useful to you?

1. Did the educators/DREAMS staff refer you to any services? If yes, please describe the referral process.
2. In your experience with the DREAMS program, do you feel comfortable/safe in participation Have you faced any negative reactions?
3. In what ways have the DREAMS services changed your life so far?
4. Do you intend to stay in the DREAMS program? Why/not?
5. Would you recommend other AGYW to join the DREAMS program? Why?

**Thank you for participating**
